# Rare variant of *TBL1XR1* in West syndrome: A case report

**DOI:** 10.1002/mgg3.1991

**Published:** 2022-05-25

**Authors:** Yajun Shen, Meng Yuan, Huan Luo, Zuozhen Yang, Mengmeng Liang, Jing Gan

**Affiliations:** ^1^ Department of Pediatrics, West China Second University Hospital Sichuan University Chengdu China; ^2^ Key Laboratory of Birth Defects and Related Diseases of Women and Children (Sichuan University) Ministry of Education; ^3^ Cipher Gene LLC Beijing China

**Keywords:** *TBL1XR1*, development delay, epilepsy, variant, West syndrome

## Abstract

**Background:**

West syndrome (WS) is an epileptic encephalopathy (EE) that begins in children 4–7 months of age (in rare cases older than 2 years). To date, over 30 genes that have been reported to be related to WS. Reports involving the extremely rare pathogenic gene, transducin beta‐like 1‐X‐ linked receptor 1(*TBL1XR1*) are quite limited.

**Methods:**

We performed exome sequencing (ES) of family trios for this infant. We also collected and summarized the clinical data for reported heterozygous germline variants of *TBL1XR1*. Moreover, we reviewed all published cases and summarized the clinical features and genetic variants of *TBL1XR1.*

**Results:**

ES revealed a de novo variant in *TBL1XR1* [NM_024665.5: exon4: c.187G > A (p.Glu63Lys)]. This variant was classified as likely pathogenic according to the ACMG (American College of Medical Genetics and Genomics) guidelines and was verified by Sanger sequencing. Further conservation analyses revealed a high conservation among several species. There was clinical heterogeneity among all patients with *TBL1XR1*‐related West syndrome.

**Conclusion:**

Our results expand the pathogenic variant spectrum of *TBL1XR1* and strengthen the pathogenic evidence of *TBL1XR1* in West syndrome.

## Graphical abstract

Introduce another West syndrome case driven by very rare TBL1XR1 variant whose phenotype and mutation sites are different from cases previously reported.

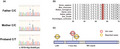

## 1 INTRODUCTION

West syndrome (WS) is an epileptic encephalopathy (EE) that begins in children 4–7 months of age (in rare cases older than 2 years). WS is characterized by infantile spasms (IS), hypsarrhythmia, an interictal electroencephalogram (EEG) pattern with irregular, high‐amplitude slow waves on a chaotic epileptic background, and neurodevelopmental delay or regression; the presence of two of these symptoms confirms the diagnosis (Hrachovy & Frost, 2013; Pellock et al., 2010; Salar et al., 2018). Over 30 genes that have been reported to be related to WS (McTague et al., 2016), most of which are extremely rare. Transducin beta‐like 1 X‐linked receptor 1 (*TBL1XR1*) is a gene reported to be associated with autistic spectrum disorder (ASD), intellectual disability (ID), and Pierpont syndrome. Some studies have also suggested that disease‐causing variants in *TBL1XR1* may contribute to genetic vulnerability to multiple neurodevelopmental psychiatric conditions. However, the evidence relating *TBL1XR1* to West syndrome is still quite limited. In this study, we collected data regarding the phenotypic and genetic variants and reviewed all reported West syndrome cases caused by *TBL1XR1*.

## 2 METHODS

### 2.1 Genetic sequencing and data analysis

Genomic DNA was extracted from blood samples of the proband and their families. xGen Exome Research Panel probes (IDT, USA) were utilized to capture the exon region following the manufacturer's recommendations, and then the libraries were sequenced on an Illumina NovaSeq 6000 platform. Raw data were mapped to the human reference genome (hg38) by the Burrows‐Wheeler Aligner (BWA) (Abuin et al., 2015), variant calling was performed by Genome Analysis Toolkit (GATK), variants were annotated by ANNOVAR, and the pathogenicity of candidate variants was evaluated according to American College of Medical Genetics and Genomics (ACMG) guidelines (https://www.acmg.net/).

## 3 RESULTS

### 3.1 Case report

**FIGURE 1:**
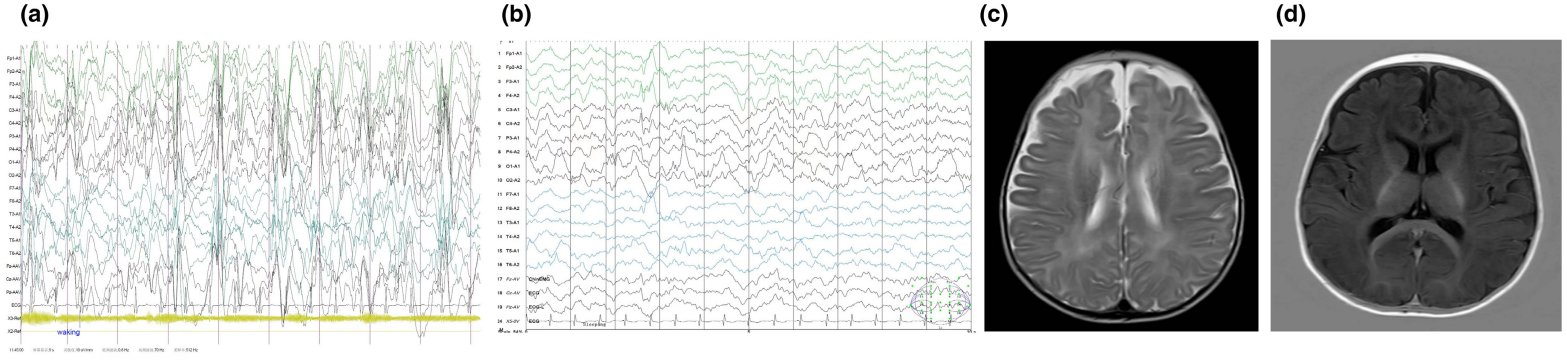
Clinical features. (a) EEG at 5 months of age showing intermittent multifocal poly spikes with irregular slow waves, indicating atypical hypsarrhythmia. Axial T2‐weighted image through the ventricles (b) EEG at 28 months of age showing great improvement in the epileptiform discharge with sporadic sharp and slow wave discharge in the left frontal, central, and occipital regions. (c) Axial fluid‐attenuated inversion recovery sequence through the basal ganglia (d) Brain MRI at 5 months of age showing mild delayed myelination.

This report concerns a 28‐month‐old girl who was born at 38 weeks and 4 days of an uneventful pregnancy to nonconsanguineous healthy parents. Her birth weight was 3700 g. She displayed head control and the ability to roll over at 3 and 4 months of age, respectively. Beyond that, she displayed no social smile or communication at 3 months of age. Upon admission at 4 months of age, she began to develop a series of epileptic seizures occurring 3–4 times a day, shortly thereafter she suffered development regression, she could not control her head and roll over, and she still showed no social smile or eye contact. She did not respond to sound or light with hypotonia of the extremities. Four small café au lait spots were found on her arms and legs that were 0.2–0.3 centimeters in size, without any neurofibromas. Her head circumference was within the normal range. No specific facial features were presented. Investigations of other organs (heart, eye, liver, kidney etc.) were negative. EEG suggested hypsarrhythmia patterns (Figure 1a). These features were consistent with a clinical diagnosis of West syndrome. Brain magnetic resonance imaging showed mild delayed myelination (Figure 1c,d). She had no dysmorphic features or stereotypical hand movements. Laboratory examination revealed that serum levels of several components were normal including lactic acid, blood ammonia, pyruvate, and β‐hydroxybutyric acid. Both blood and urine metabolic screening were normal. Neither administration of adrenocorticotropic hormone therapy for 28 days nor high‐dose vitamin B6 reduced the frequency of spasms. Therefore, we attempted to control the seizures with topiramate, which also failed. We consequently began a trial of vigabatrin. Finally, she was seizure‐free 1 month later on a combination therapy of vigabatrin (100 mg/kg/d) and topiramate (8 mg/kg/d). The child had no other skin problems, giant cell astrocytoma, cortical tubers, or subependymal nodules. Furthermore, we observed no kidney, heart, eye, or lung lesions. By the last follow‐up of 28 months old, she had been seizure‐free for more than 20 months. She could only sit without support, and she spoke no words. Her Gesell Developmental Scale score was 30. Repeated EEG monitoring showed great improvement in the epileptiform discharge with sporadic sharp and slow wave discharge in the left frontal, central, and occipital regions (Figure 1b).

### 3.2 Genetic findings and literature review

**FIGURE 2:**
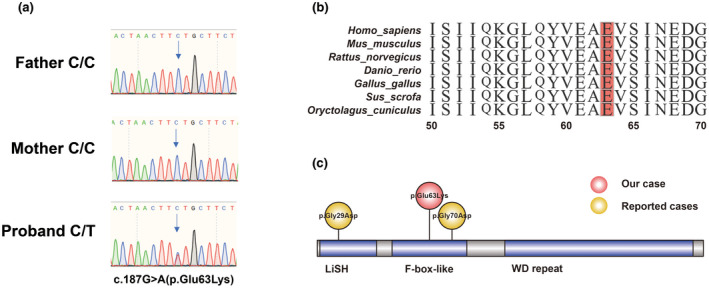
De novo *TBL1XR1* variant. (a) De novo variant of the c.187G > A (p.Glu63Lys) in the proband family. (b) Conservation analysis of p. Glu63Lys among multiple species, variant amino acids are highlighted in orange color. (c) Two previously reported variants (p.Gly70Asp and p.Gly29Asp) are shown as orange balls, and our patient is represented by a red ball.

G‐banded karyotyping (46, XX) and 2.7 M pathological copy number variation array (Affymetrix, Santa Clara, CA, USA) of the proband showed normal results. Exome sequencing uncovered a de novo variant in the child: *TBL1XR1* [NM_024665.5: exon4: c.187G > A (p. Glu63Lys)]. The variant was confirmed by Sanger sequencing (Figure 2a), absent in ExAC, gnomeAD, and 1000genome, predicted as damaging by several protein prediction tools (SIFT: D; Polyphen2: D; MutationTaster: D), so it is regarded as likely pathogenic according to ACMG guidelines (Table 1). Further conservation analysis confirmed that this amino acid was highly conserved across species (Figure 2b). We reviewed all published cases and summarized the clinical features and genetic variants of *TBL1XR1* (Table 2), and found that all cases had developmental delay. In addition, our patient and Saitsu et al.’s patient (Saitsu et al., 2014) exhibited delayed gross motor skills (both variants are located in the F‐box‐like domain), while the patient in Alison et al.’s study (Muir et al., 2019) showed hyperactive behavior and attention deficit disorder (variant located in the LiSH domain).

**TABLE 1:** Pathogenicity analysis of the variants in *TBL1XR1*

Gene	Variant	Inheritance	MAF			SIFT	Polyphen2	Mutation taster	Evidence	ACMG Category
			ExAc	gnomAD	1000 genome					
*TBL1XR1*	c.187G > A	De novo	NE	NE	NE	D	D	D	PS2 + PM2_supporting+PP3	Likely Pathogenic
	p.Glu63Lys									

**TABLE 2:** Clinical features summary of *TBL1XR1*‐related diseases

	Age of seizure onset	Types of syndrome	Initial EEG	MRI	Developmental Delay	Behavioral issues	Genetic analysis	Variant
Our patient	12 m	West syndrome	Hypsarrhythmia	Mild delayed myelination	+	−	ES + CNV	NM_024665.5: c.187G > A, p. Glu63Lys
Saitsu et al. (2014)	5 m	West Syndrome	Hypsarrhythmia	Mild cerebral atrophy	+	Autistic Features	ES	NM_024665.4: c.209G > A,p. Gly70Asp
Muir et al. (2019)	6 m	West syndrome	Hypsarrhythmia	Mild delayed myelination; poor white matter development; mild vermis hypoplasia and thin corpus callosum (6 m, 30 m, 5y)	+	Hyperactive behavior and attention deficit disorder	ES	NM_024665.4: c.86 G > A,p. Gly29Asp
Tabet et al. (2014)	−	−	Normal	Normal	+	Psychomotor instability, short attention span, trichotillomania, reactional aggressive behavior but no ASD	SNP array	1.6 Mb deletion in 3q26.31q26.32 region: arr[hg19] 3q26.31q26.32 (175,507,453–177,095,072) × 1
Heinen et al. (2016)	−	Pierpont syndrome (6/6)	N/A	Central atrophy (3/6), enlarged ventricles (2/6), choroid plexus papilloma (1/6)	1	−	ES	NM_024665.4: c.1337A > C, p. Tyr446Cys
Zaghlula et al. (2018)	−	Rett syndrome	Normal	Mild prominence of the perivascular spaces and borderline thinning of the body of the corpus callosum	+	Rett features	ES	NM_024665.4: c.1108 G > A, p. Asp370Asn
Pons et al. (2015)	−	−	Normal	Normal	1	−	aCGH	708 kb‐microdeletion on chromosome 3q26.32: arr [hg19] 3q26.32 (176,780,822*2,176,221,801‐176,929,584* 1,176,983,401*2)
Kahlert et al. (2017)	−	Pierpont syndrome	N/A	N/A	+	N/A	ES	NM_024665.4: c.1337A > G, p. Tyr446Cys
Slavotinek et al. (2017)	−	Pierpont syndrome	N/A	Arnold Chiari malformation	+	N/A	ES	NM_024665.4: c.1337A > G, p. Tyr446Cys
O'Roak, Vives, Girirajan et al. (2012)	−	Autism (2/2)	N/A	N/A	0.5	N/A	Massively multiplex‐targeted sequencing	NM_024665.4: c.845 T > C, p. Leu282Pro
Riehmer et al. (2017)	−	3q26.32 microdeletion syndrome	N/A	Dandy Walker malformation (1/4)	4/4	Autism spectrum disorders (2/4)	array‐CGH	309 Kb microduplication of genetic material of chromosome 3q26.32: arr [hg19] 3q26.32(176,648,502– 176,957,675) × 3 & a 521 Kb microduplication arr [hg19] 3q26.32(176,627,832–177,149,304)x3
Ismaili‐Jaha et al. (2021)	−	Pierpont syndrome	N/A	relatively cortical and central hypoplasia of the brain, small frontal lobes as well as minor dilatation of the third and lateral ventricles	+	Stereotypic behavior	ES	NM_024665.4: c.1337A > G, p.Tyr446Cys
Tesarova et al. (2022)	−	Pierpont syndrome	N/A	small pituitary gland and hypoplasia of the corpus callosum	+	N/A	ES	NM_024665.4: c.1337A > G, p.Tyr446Cys
Arroyo Carrera et al. (2021)	−	Pierpont syndrome and autism	Normal	Arnold Chiari malformation	+	N/A	ES	NM_024665:c.710G > A, p.Gly237Asp
Aguilera et al. (2021)	N/A	Angelman syndrome‐like	N/A	N/A	+	Stereotypic and aggressive behavior	ES	NM_024665.5: c.1000 T > C, p.Cys334Arg

## 4 DISCUSSION

The *TBL1XR1* gene is located at 3q26.32. It encodes the protein transducin‐beta‐like‐1 X‐linked receptor 1, which contains a LisH domain (Lis1 homology domain), an F‐box‐like domain at the amino terminus, and seven WD40 repeats at the carboxy‐terminus (Zhang et al., 2006). Related phenotypes include intellectual disability, Pierpont syndrome, autism spectrum disorders, and intellectual disability with dysmorphism (O'Roak, Vives, Fu et al., 2012; Pons et al., 2015; Saitsu et al., 2014; Tabet et al., 2014). *TBL1XR1* is essential in the activation of Wnt‐β‐catenin signaling pathways, which is an indispensable factor in the functioning and activity of β‐catenin–Tcf‐mediated Wnt signaling (Choi et al., 2011; Li & Wang, 2008). *TCF4* is an essential mediator of Wnt signaling. Pathogenic variant of *TCF4* has been revealed to be related to Pitt–Hopkins Syndrome which is characterized by severe intellectual disability, seizures, and stereotypic movements (Zweier et al., 2007). These findings indicate that the β‐catenin–Tcf‐mediated Wnt signaling pathway is vital for brain function normalization. Moreover, a 5‐year‐old Japanese girl with West syndrome features was identified to have a de novo heterozygous c.209G‐A transition (c.209G‐A, NM_024665.4) in the *TBL1XR1* gene, which results in a gly70‐to‐asp (G70D) substitution at a conserved residue in an F‐box‐like domain (Saitsu et al., 2014). The interaction of *TBL1XR1* and *SMRT*, a corepressor of nuclear hormone receptors, is influenced by the F‐box‐like domain of *TBLR1* (*TBL1XR1*) (Zhang et al., 2006). Therefore, this report implied that the pathogenic *TBL1XR1* variant may cause West syndrome features (Saitsu et al., 2014). In addition, there was a second case reporting an individual with West syndrome who had a de novo p.Gly29Asp (NM_024665.4:c.86 G > A) variant in the N‐terminal LisH domain of *TBL1XR1* (Muir et al., 2019).The LisH domain is required for oligomerization, transcriptional repression, and binding to hypoacetylated H2B and H4 (Yoon et al., 2005). Deletion of the LisH domain decreases the half‐life of the *TBL1XR1* protein and results in its translocation from the nucleus to the cytoplasm (Gerlitz et al., 2005). This may consequently cause West syndrome.

## 5 CONCLUSION

In this patient, we describe a de novo *TBL1XR1* variant that may lead to West syndrome via the Wnt signaling pathway. To the best of our knowledge, our patient is the third patient with *TBL1XR1* driving West syndrome. We reviewed the clinical features of the limited examples of West syndrome being driven by the *TBL1XR1* variant (Table 1). Our report strengthens the etiology of *TBL1XR1* as a West syndrome pathogenic gene.

## AUTHOR CONTRIBUTIONS

**Yajun Shen & Meng Yuan:** Conceptualization, Methodology, Data mining, and Writing‐ Original draft preparation; **Huan Luo:** Writing‐Original draft preparation, Methodology; **Zuozhen Yang & Mengmeng Liang:** Software, Data mining, and Investigation; **Jing Gan:** Supervision, Writing‐ Reviewing, and Editing.

## FUNDING INFORMATION

This work was supported by the National Science Foundation of China (No. 82071686), the Grant from Science and Technology Bureau of Sichuan province (No. 2021YFS0093), and the Grant from Research Fund of West China Second University Hospital (No. KL115, KL072).

## CONFLICT OF INTEREST

The authors declare no conflict of interest.

## ETHICAL COMPLIANCE

This study was approved by the Ethics Committee of West China Second University Hospital of Sichuan University. Informed consent was obtained from the proband and their families. Clinical manifestations, EEG, other clinical results, and gene variations were investigated.

## Data Availability

The data that support the findings of this study is available in ClinVar database, the accession number is SUB11348360.
